# A qualitative exploration of NHS-staff social identification with mindfulness in-groups and engagement with mindful practices

**DOI:** 10.1371/journal.pone.0331196

**Published:** 2025-10-23

**Authors:** Daniel Cullen, Kate Cavanagh, Clara Strauss

**Affiliations:** 1 School of Psychology, University of Sussex, Brighton, United Kingdom; 2 Sussex Partnership NHS Foundation Trust, Worthing, West Sussex, United Kingdom; Private University Schloss Seeburg: Privatuniversitat Schloss Seeburg, AUSTRIA

## Abstract

**Background/ objectives:**

Mindfulness practice has been widely adopted by healthcare staff, being grounded in evidence and supported by national guidelines. However, certain fundamental factors are not well understood, including attitudes towards mindfulness in-groups. The aim of this project was to explore the nature of social identification with mindfulness in-groups in relation to engagement with mindful practices.

**Methods:**

Twenty healthcare staff from three healthcare Trusts were recruited to engage in semi-structured interviews of one-hour duration. Transcripts of these interviews were then subjected to reflexive thematic analysis.

**Results:**

Social identification was simultaneously rich and fluid, with some tentative evidence of positive boosts to psychological engagement tempered by dissonance about current physical engagement levels. Healthcare and mindfulness identities complemented, in that being aided doing, aligning values and aspirations, although clashes also presented through culture, depending on one’s sense of connection/disconnection.

**Discussion/ conclusions:**

Employers and trainers could consider implementing strategies to enhance social identification with mindfulness in-groups. Further research should also explore whether strength of social identification translates into improved engagement with mindful practices.

## Introduction

Healthcare services around the world are still recovering from the global impact of Covid-19. Aside from the serious consequences felt by patients and families, there is growing recognition of the detrimental effects on healthcare staff [1–3]. In the UK’s National Health Service (NHS), as elsewhere, even before the pandemic, healthcare staff were some of the most stressed workers in the country [4]. During the pandemic, regardless of healthcare profession or role, the prevalence of common mental disorders elevated in parallel with patient deaths, and reports of anxiety increased over time [5]. In one observational study, approximately one third of healthcare staff reported moderate to severe depressive symptoms [6]. Further evidence suggested a three-fold rise in risk of burnout in healthcare staff compared to non-healthcare staff [2]. As highlighted by Lamb and colleagues [7], the number of stressors that both clinical and non-clinical staff have faced is extraordinary: Death of patients/colleagues at work and loved ones back at home, risk of personal infection, lack of personal protective equipment, changes in work patterns, suboptimal working conditions, and moral injuries. The worst of the pandemic may have passed, as have national lockdowns, but there has been little by way of reprieve. Healthcare staff today face increased workloads and backlogs for work deferred when prioritising Covid-19 [8,9]. The number of staff who are thinking of leaving their roles is on the rise, with commonly cited reasons being wellbeing concerns, need for time off, feeling undervalued, too much pressure, exhaustion, and under-staffing [10–12]. Stress, anxiety, depression, and other psychiatric disorders recently ranked the most common causes of sickness absence, accounting for almost a quarter of all cases [13]. Hence, it is imperative that all effective means of support are made available to healthcare staff, to safeguard their mental wellbeing as the NHS recovers.

Mindfulness could have a vital part to play in supporting healthcare staff with their mental health and wellbeing. It can be defined as awareness that comes from paying deliberate and non-judgemental attention to thoughts and feelings that arise in the present moment [14]. Mindfulness meditation practice descends from Eastern traditions and gained popularity in the West in the 1980s with the development of mindfulness-based interventions (MBIs) [15]. The first MBI to come to prominence was Mindfulness-Based Stress Reduction (MBSR), developed in the late 1970s as an intervention for adults experiencing chronic stress, pain, and longstanding physical health conditions [16]. Mindfulness-based cognitive therapy (MBCT) is a more recent adaptation of MBSR and cognitive therapy, developed for adults vulnerable to depression [17]. Both interventions include guided mindfulness practices and teacher-led discussion about learning from practice. They are delivered in 8-weekly in-person group sessions of at least two hours duration plus a homework component equating to 45 minutes of mindfulness practice and other exercises per day. However, since the inception of MBSR, MBIs now come in many forms that share common principles and components [18], including guided and unguided self-help interventions supported by books, online courses and smartphone apps. Much of their appeal is their strong evidence-base for effectiveness in improving a wide range of outcomes in clinical and non-clinical populations and workplace settings [19,20]. Regarding healthcare staff specifically, recent meta-analyses of randomised controlled trials (RCTs) consistently show positive reductions in stress, anxiety, and depression for staff receiving a range of MBIs when compared to a range of control conditions, with medium effect sizes [21–23]. Beneficial effects of digital MBIs (i.e., MBIs that can be accessed using apps/technology/online) have also been found, although effect sizes appear to be smaller. For example, in a recent RCT of unguided use of the digital app in NHS staff, Headspace was shown to improve stress and a range of secondary outcomes including depression, anxiety, and wellbeing in healthcare staff, when compared with an active control with significant but small effects [24]. Based on the strength of such evidence, national guidelines in England advocate the provision of mindfulness training (in groups and/or online) for staff of all organisations, including healthcare staff [25].

Whatever the format of the MBI, engagement with the intervention, and regular mindfulness practice in particular, are thought to be key to their potential benefits [26]. As highlighted by Banerjee and colleagues [27], however, engagement with MBIs may differ from engagement with other well-known health behaviours, since rather than just physically participating (the “doing” aspect), participants are invited to engage psychologically and cultivate a different way of being. Alongside physical engagement, they operationalise psychological engagement as: motivation to assign time, intention to practice further, commitment to being more mindful in daily life, believing in the potential benefits, and being present to the therapeutic relationship [27]. In a prior qualitative study of barriers and facilitators to engagement with mindfulness-based self-help (MBSH) in NHS staff, numerous themes were found to support this definition of psychological engagement, such as “attitude towards engagement” and “motivation to reduce stress”, or “perceived effects of mindfulness on mental health and wellbeing” [28, p.1657]. Taking the fifth criterion, presence to the therapeutic relationship was evidently an important aspect of engagement in prior qualitative studies across a range of populations in receipt of group-based MBIs, for example when group members came together to normalise experience and reduce isolation [29–31]. Banerjee and colleagues argue that presence to the therapeutic relationship might even be achieved with MBSH. After all, this is about mental presence, not physical presence. At the same time, few studies have gone further to assess participant perceptions of themselves as part of a wider mindfulness collective, or what this could mean for psychological engagement. For instance, a qualitative study derived from focus groups of healthcare staff working on an adolescent mental health unit found that “mutual mindfulness experience” helped foster a shared identity/ in-groups via shared empathy among staff, yet, only the physical MBSR group was considered within this organisational context [32, p.6]. This is a potentially important gap when considering the many ways that individuals can seek to cultivate mindfulness, some of which do not require physical presence; examples include digital self-help apps that may be self-guided only (i.e., where a sense of community happens solely in the mind), online/ remotely delivered MBI’s/drop-ins, forums and/or online communities, or even the growing popularity of mindfulness as a movement, beyond in-person mindfulness groups.

As per Tajfel and Turner’s Social Identity Theory [33], social identification with mindfulness in-groups (e.g., the attended mindfulness group and/or the wider mindfulness community) could be a contributing factor in psychological engagement with mindfulness. Social identification is the term used in Social Identity Theory to refer to “the positive emotional valuation of the relationship between self and ingroup” [34, p.599]. Haslam and colleagues highlight just some of the ways that Social Identity Theory has been applied to health and wellbeing in a special issue on this topic, including developments by Turner in Self-Categorisation Theory (see Haslam et al., 2009 for overview). From the broad body of evidence synthesized therein, in-groups should be viewed as internalized group memberships that serve a variety of important functions – they provide a sense of belonging, meaning, worth, means of safety and emotional support, imbue a sense of purpose, and contribute to positive psychological health, among other benefits. Indeed, identification with in-groups is linked to sense of self and, crucially, motivation to engage with other members and the normative practices of that in-group, via shared social influence [35,36]. In one study of nurses, for example, social identification with the nursing in-group was an independent predictor of flu vaccination take-up by nurses, even when health-related knowledge was factored in [37]; subsequent mediation analysis showed the mechanism of action to be professional duty, suggesting a mediating role for internalization of group norms on group member action to protect patients. In the case of healthcare staff, it seems intuitive that both healthcare groups and mindfulness groups might positively contribute to social identity, but that healthcare in-groups have a greater emphasis on doing, whereas mindfulness is quintessentially about being. Even though both groups might contribute to identity while holding some opposing values, acquisition of in-group status is not likely to be synchronous, and even positive changes to social identity might be experienced negatively as “loss of psychological “footing”” as individuals adjust to their new identity (35, p.5). It may be that healthcare and mindfulness identities are not opposed at all, perhaps working in synergy in ways previously unexplored. Either way, Tajfel and Turner (33, p.40) postulate that the individual is constantly striving towards a positive identity and that maintenance of identity depends on “favorable” comparisons but also distinctiveness from out-groups. The consequences of a mismatch could in principle lead to disengagement from the unsatisfactory in-group, or renewed efforts to increase its positive distinctiveness.

Social identification may hold one of the keys to a better understanding of psychological engagement with mindful practices, as healthcare staff struggle to physically engage with mindful practices against a unique backdrop of NHS stressors and adaptations. The pre-registered aim of this study was “To better understand staff attitudes to groups and the role of social identification with mindfulness in relation to psychological engagement”. The objective was “To qualitatively explore social identification with mindfulness and psychological engagement, in healthcare staff in different roles and at different levels of seniority”. Formulated as research questions, we asked: 1) how might NHS staff socially identify with mindfulness (i.e., groups or communities), and 2) how might this relate to psychological engagement? In keeping with Social Identity Theory, moreover: 3) how might cooccurring healthcare identities/in-groups complement or clash with mindfulness identities/in-groups? To our knowledge, this is the first study to examine social identification with the full spectrum of mindfulness in-groups and psychological engagement in healthcare staff.

## Method

### Design

The present qualitative study was a sub-study of MindArise, a longitudinal mixed-methods investigation examining mindfulness and response in NHS staff with experience of mindfulness across 53 NHS sites in England (https://www.isrctn.com/ISRCTN57729713). Recruitment ran from 01 September 2022 to 28 February 2023, with three waves of data collection at baseline, 3 months and 6 months. Qualitative interviews ran in parallel with the first phase of longitudinal data collection and were intended to provide a timely overview/understanding of staff engagement with mindfulness, to explore the group-based contexts that engagement may be influenced by, and to inform questions related to potential associations between social identification and outcomes at Time 3. Qualitative interviews were conducted by the first author, a PhD student at the University of Sussex with former experience in mental health nursing (prior to lapsing registration and ceasing to work as a nurse), clinical trials research, motivational interviewing, and motivational enhancement therapy. Supervision was provided by Clinical Psychologists, Professor Kate Cavanagh and Professor Clara Strauss, both of whom having training in mindfulness-based approaches. A small group of healthcare staff were also invited to offer their perspectives on participant-facing materials before the study went live as part of ongoing stakeholder activity for the wider MindArise investigation.

### Participants

Eligible participants needed to have current or previous experience of formal and/or informal mindfulness practice as defined in the participant information sheet, be aged 18 or over, currently employed as healthcare staff by an NHS Trust or Primary Care provider in England (clinical or non-clinical roles, full-time or part-time, in training or voluntary permitted), not currently on long-term sickness absence which we defined as 4 + weeks of continuous sickness, with sufficient proficiency in English, and access to a computer/ electronic device, to be able to complete the online questionnaires, which were presented in English.

Thematic saturation and information power were both considered when determining sample size for this study. On the one hand, prior studies found 12 to the be minimum number of interviews required to reach thematic saturation, or the point where no new themes occur [38,39]. However, others are sceptical of saturation claims and the lack of transparency around sample-size sufficiency [40]. Counter to saturation, Malterud and colleagues [41] suggest that sample size be based on information power, as indicated by how narrow or broad a given study is in its study aims, sample specificity, theoretical background, dialogue quality and analysis strategy. The guiding principle of this concept is that the more information a given sample has to offer, the fewer participants are needed. As the present study was deemed to occupy the mid-point on most of these continuums – having 1) specific aims, 2) healthcare staff all with experience of mindfulness, 3) established theory, 4) an interviewer with experience in the NHS and interviewing, 4) cross-case analysis – a mid-range sample of 20 participants was proposed, with provision to stop at 12 in the unlikely event of saturation.

In total, 20 healthcare staff were recruited from the wider MindArise investigation, covering the full range of NHS roles listed on the Health Careers website [42]. Participants came from Medicine and Nursing, Occupational Therapy, Physiotherapy, Psychology/IAPT, Speech and Language Therapy, Social Work, Clinical Research, Medical Recruitment, Executive Boards, and Advocate Services. NHS experience ranged from less than one year in entry-level roles to senior roles with 35 years’ experience (M = 15.5, SD = 10.55). Number of years since participants first practiced mindfulness (allowing for intermittent patterns of formal and informal practice that sometimes equated to very little practice experience) ranged from three years to twenty-nine years (M = 8.7, SD = 6.06). At the time of interview, 85% of participants were formally practicing either consistently or intermittently using a range of formats (e.g., mindfulness groups, digital mindfulness apps, and unguided mindfulness meditation). Ages ranged from 25 to 60 (M = 42.75, SD = 9.29), and 75% of participants were female. Eighty percent of participants identified as White, jointly followed by 10% Asian British, and 10% Mixed/Multiple Ethnic Groups. Sixty percent of participants identified as having no religion, followed by 15% Buddhist, 10% Christian, and 5% Muslim, Sikh, and Jewish faiths respectively. Sample demographics broadly matched NHS workforce population demographics [43–45].

### Procedure

Due to different NHS Trusts joining the wider MindArise investigation at different timepoints, participants for this sub-study were recruited from three NHS Trusts offering community and/or mental health services in Sussex, Lincolnshire, and London. Advertising was via online adverts, intranet adverts, email distribution lists, posters/leaflets, and word of mouth. Prospective participants who followed links to this study were presented an electronic participant information sheet and consent form to complete online. Those who selected the optional opt-in for interview were then invited to take part in interviews on a first come first served basis. A convenience sampling strategy was used due to the project’s time-sensitivity, with all interviews completed over approximately six weeks. Consenting participants were invited to book a convenient timeslot online using the Calendly scheduling automation platform [46]. Where participants did not attend for their scheduled interviews, two reminder emails were sent to reschedule, after which interviews were either completed or reallocated to the next available participant.

### Interviews

Twenty interviews were conducted by the first author online using the Zoom video conferencing platform [47], with a planned duration of 60 minutes. Actual duration ranged from 43 minutes 44 seconds to 86 minutes 43 seconds (M = 61 minutes 36 seconds). The interview schedule (see S1 File) was semi-structured to allow for in-depth conversations and flexibility when exploring participants’ thoughts and beliefs in relation to the study’s research questions. The conceptual basis for some questions was taken from Prochaska and DiClemente’s [48] transtheoretical model of behaviour change, which presupposes a cycle of stages from pre-contemplation to contemplation, preparation, action, and maintenance of a given health behaviour. Aspects of the interviewer’s prior experience with motivational interviewing were also employed, such as reflective listening, although no attempts were made to elicit insight or behaviour change.

The semi-structured interview questions were predominantly open-ended to encourage full, unfettered reflection and sharing of information. Strategic use of closed-questions was also planned for filter questions when setting up open-questions, clarifications of prior responses, navigation away from participant drift, and as part of the reflective process when checking for accuracy or building rapport. Example questions included: “What is your impression of other people who practice mindfulness?”; “What is your impression of mindfulness teachers?”; “What would you say about mindfulness to someone who was considering it?”; “Do you personally identify with mindfulness groups, movements or group members in any way?”; and “How does this association make you feel?”.

Each interview was framed by a 1-minute check-in to remind participants not to use names or other identifiers of people or places when the interview commenced. During this time, participants were also reminded that interviews would be recorded using a Dictaphone for transcription purposes (not video-recorded on Zoom) and that recordings would be permanently deleted on completion of the study. Recordings began when participants indicated they were happy to continue. A two-minute debrief was conducted by the interviewer after recording stopped, to check-out and thank participants for their time.

Resulting interview recordings were played, replayed and transcribed in true verbatim by the first author. Stutters, false starts, and filler words have been removed from the present paper to prevent redundant information from impacting readability. The first author checked transcripts against recordings to ensure accuracy.

### Planned analysis

Reflexive thematic analysis, according to Braun and Clarke’s method [49], includes: 1) familiarisation with the data by reading and re-reading transcripts and re-listening to audio recordings, 2) identifying and collating codes that represent important data features, assisted by use of NVivo software in the present study [50], 3) identifying data patterns or candidate themes, 4) checking for congruence of themes and codes and removing and developing these accordingly in an iterative process, 5) refining the scope of themes and giving them names, and 6) writing up the paper [49]. All steps were initially undertaken by the first author, including the process of transcription to help with data immersion, with subsequent steps discussed in supervision with authors 2 and 3. A flexible top-down (deductive) and bottom-up (inductive) analysis was employed in respect of research questions that were partially grounded in theory. Inductive analysis was the most dominant approach, however, due to a deficit of literature in this given field. Specifically, deductive analysis of the data was applied when seeking to determine pre-specified conditions for social identification, as per the theory. In contradistinction, inductive analysis took two forms: 1) Exploration of potential themes that arose from immersion in the data when conducting and subsequently transcribing the interviews. The first author noted any prominent patterns and subsequently explored those patterns of meaning as presented in relevant codes. 2) The body of codes was subjected to further analysis for potential patterns that were not immediately obvious from prior immersion in the data. Credibility checks of initial codes and themes were conducted by second and third authors, making use of detailed code lists and transcript extracts during a series of thematic workshops attended by all three authors as part of stage five of Braun and Clarke’s method. Minor changes to some of the theme names and model structure were agreed but otherwise there were no disagreements on codes/themes.

A critical realist approach was adopted by the first author, assuming that some degree of reality can be understood through human experience and interpretation [51], while additional steps were taken to ensure rigour and reflexivity. “Bracketing” as a method lacks consensus in qualitative research, but broadly denotes conscious means of researcher reflexivity on their own assumptions, values and/or other subjective preconceptions [52]. To this end, the first author kept a reflective journal, comprised of one in-depth reflective account prior to conducting any interviews, followed by analytical memos conducted post-interview (x20), and reflective accounts conducted during the analysis. Bracketing interviews and/or alternative triangulation methods that employ external researchers were not considered appropriate or pragmatic in the available time. Notably, the in-depth reflective account took the form of a reverse interview using the same interview schedule presented to participants. By answering these questions in turn, the first author became aware of strong beliefs about the need for greater understanding of self, others and the world, values of insight, mastery and compassion, and personal aspirations of being more mindful formally and informally. General assumptions included cynicism around professional demarcation, positive views of mindfulness practitioners and teachers in general, but negative views of practitioners or teachers who do not practice what they preach. Reflective discussions were subsequently held with second and third authors, thus supporting receptiveness and curiosity to the experiences of interviewees in the co-construction of meaning.

## Results

**Fig 1:**
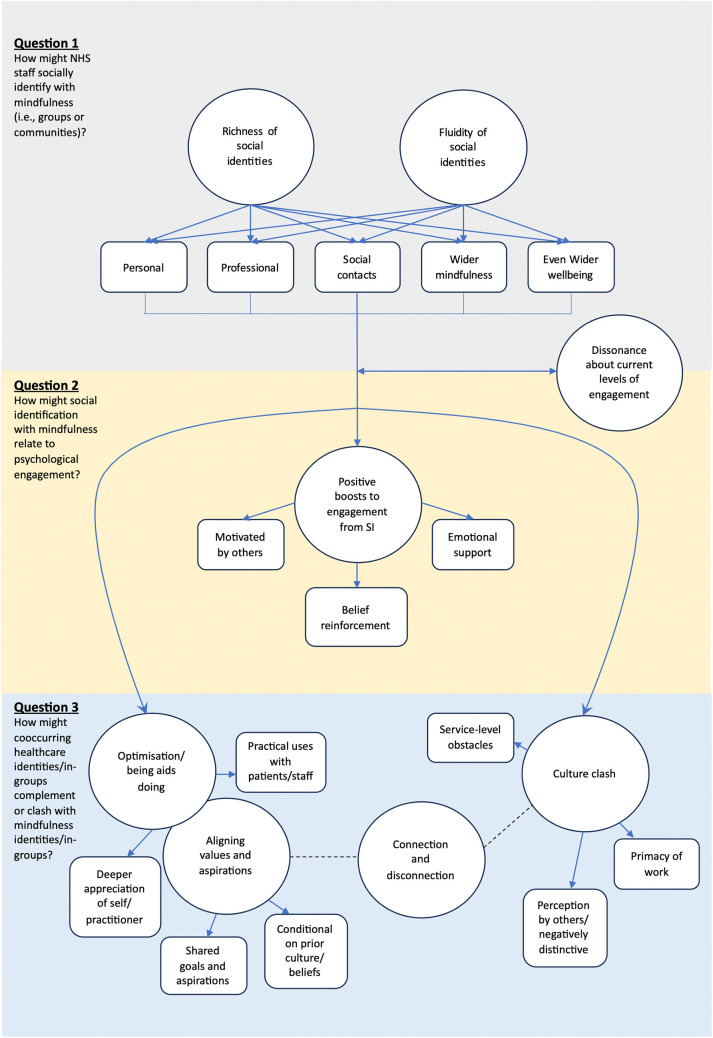
Social identification in relation to psychological engagement in healthcare staff.

Eight themes resulted from the analysis; these are described below (themes and subthemes in bold), with the proposed model for themes and subthemes presented in Fig 1. Arrows connecting themes and subthemes represent nesting only, whereas arrows connecting themes to other themes represent their observed correlations (their inter-relatedness or apparent influence on each other), as grounded in some but not all interviewee responses and the progression of their ideas and experiences. Relationships of causality should not be inferred.

### Richness of social identities

Social identification with mindfulness identities/in-groups was rich and nuanced, with the majority of interviewees expressing social identification with one or more mindfulness group concepts to varying degrees. For example, “it is wrapped into and has a defining component of my professional identity and my personal identity” (Interviewee 19). However, some participants identified with mindfulness on a personal level only, in which case, some key elements of identification were present in the absence of a social context.

#### Personal.

The majority of interviewees identified with mindfulness as part of their personal identity, irrespective of social extension to practicing others. Being someone who practices mindfulness was perceived as positively distinctive and important to one’s sense of self. According to one interviewee, “I like being part of it. I like [slight laugh]. I like knowing this, it’s kind of like a secret, isn’t it. But not everybody knows about it” (Interviewee 21). According to another:

For me it’s a more, it creates more positive sense of self. It’s the side of me that I like most, I think, when I practice mindfulness and when I’m in that headspace. And yeah, cause like I said, I just think I become a better person for it, so. Yeah, I think I’d like to do it more, I’d like to be able to become that person more (Interviewee 02).

To some extent, personal identification was about embodiment of mindfulness principles, and for some people that was sufficient:

what I’m hoping is that I actually embody different aspects of mindfulness. So I think I’m hoping that I embody this non-judgemental stance and, as I kind of mentioned, that sense of compassion for self, for myself and for others (Interviewee 12).

At the same time, others voiced the desire to move beyond mindfulness at the personal level, that is, to move towards mindfulness groups/community:

I’d like to expand it into something more, and that would be where the community would come in for me I think, and sort of being more able to share that with other people (Interviewee 13).

#### Professional.

Some interviewees identified with mindfulness as part of their professional identity. Interestingly, this was comprised almost exclusively by interviewees from psychological professions that use mindfulness at the service level. According to one interviewee, “I tend to I think gravitate towards subgroups within psychology that emphasise the use of mindfulness” (Interviewee 18). Another interviewee stated, “I’m associating it with work rather than my own personal improvement” (Interviewee 14). This even affected their behaviour, i.e., “doing mindfulness in the same way the rest of the team is and using the same kind of language” and “trying to do the right thing or to impress my team, to give this impression of being good at mindfulness for my team (Interviewee 14). Being someone who practices mindfulness in a professional context was likewise perceived as positively distinctive:

at work there’s more of an understanding around mindfulness and I will meet more people that do have a personal mindfulness practice and value it. Outside of work, no, I would think that some people probably think it’s a bit unusual (Interviewee 24).

Highlighting importance, another interviewee stated, “I don’t think you devote twenty-odd years to something that didn’t feel has become something quite integral” (Interviewee 19).

#### Social contacts.

Over half of interviewees identified with people they met who practiced mindfulness, in-person or online. For example, one interviewee said, “I did strongly associate it with the groups that I did on Zoom, when they were running (Interviewee 6). Another interviewee explained, “I generally have a lot of time for people that I’ve met that practice mindfulness” (Interviewee 27), adding, “I’m slipping into the thinking that I get with some other types of therapy that I’m very dismissive of, but I don’t feel like that about people that practice mindfulness”. Some participants recalled important individuals such as close friends who inspired them to pursue mindfulness, or in one case, “my [relative] talked about it as well and I think [they] read up on it a bit more, so yeah I did have this idea that mindfulness is something, yeah quite beneficial, it’s something quite good” (Interviewee 12). These associations were relatively distinct from associations with the wider mindfulness community, as expressed by one interviewee, “I probably don’t identify with everyone necessarily in the entire mindfulness community” (Interviewee 18), and then, “But definitely, you know, smaller groups within that […] I probably identify with quite strongly” (Interviewee 18). There was positivity and importance placed on these relationships, for example, “that feeling that there’s some other people there that can relate. I think that’s massively powerful” (Interviewee 10).

#### Wider mindfulness.

Over half of interviewees also identified with the wider mindfulness community, including unknown individuals. One interviewee referred to this community as true-woo:

let me define woo woo, a true-woo. So the woo-woo is like, that is properly bonkers, but a whole section of stuff that I dismissed as properly bonkers turns out when you do the science, and you take a different approach, […] then you start to think there’s something going on here. That’s my tribe, the people who link science with this type of subjective practice (Interviewee 22).

Expanding on the source, “I haven’t met them but then you meet people virtually don’t you […] through their YouTubes, channels and all the rest. Because this is about evidence, you meet them through their writing” (Interviewee 22). There was even a degree to which whole philosophies were identified with, i.e., “a relationship with the concepts of Buddhist philosophy and mindfulness” (Interviewee 24), such that, “I think they influence your values” (Interviewee 24). Across interviewees, there was a sense of warmth and positivity towards the wider mindfulness community, described by one interviewee as, “quite exciting, really interesting, very meaningful” (Interviewee 19). Moreover, there was a hypothetical dimension to positive identification, for example, “if I was going somewhere where I knew there was somebody who did mindfulness, I’d probably gravitate towards them” (Interviewee 36), and “I see it very much as a therapeutic relationship to a community that I can’t quite get to yet” (Interviewee 22).

#### Even wider wellbeing.

Some interviewees identified with the even wider wellbeing community, including but not limited to mindfulness. One interviewee listed “yoga and nature, and outdoors and walking” (Interviewee 30). Indeed, several interviewees talked about their affinity with a yoga group and how this espouses mindfulness-based principles: “one of my most important group memberships is the yoga class I go to, for example, and I go every week. And that’s really important (Interviewee 31). This interviewee explained:

I do count the hour and a half of yoga that I do as mindful meditation, because it is. It’s with movement. I’m very much noticing my mind in that space, that it certainly is something I couldn’t be without (Interviewee 31).

Ultimately, people connected strongly with others in yoga groups, for example:

Even if you don’t really know them very well. I guess you have maybe an assumption [slight laugh] that you probably share similar values and maybe you share similar outlooks on life. So I think there is that feeling of identification (Interviewee 18).

### Fluidity of social identities

Social identification was by no means consistent, being notably fluid in how positive attitudes could come and go. A few interviewees claimed to identify with one or more of the group concepts shown above, on the one hand proffering positive views about identification with mindfulness identities/in-groups while subsequently contradicting said views in a manner reminiscent of possible ambivalence. For example, “that’s a big association for me” (Interviewee 30) followed from, “that association, I feel like it changes. I think at the moment it doesn’t feel strong”. Consequently, there was movement in attitudes.

### Positive boosts to engagement from social identification

There was a pattern of positive boosts to engagement from social identification. As highlighted by one interviewee, “no-one wants to feel alone truly in what they do. I think it’s nice to know that these boosts belong to everybody” (Interviewee 13). About half of interviewees identified ways that social identification with mindfulness identities/ in-groups helped them with psychological aspects such as motivation, beliefs, and intention to maintain a mindfulness practice. It is important to stress, though, that experiences of positive boosts to engagement did not necessarily map onto higher levels of mindfulness practice.

#### Motivated by others.

Some interviewees said that mindfulness identities/ in-groups either helped or would help their motivation to engage with mindfulness practices. A few interviewees talked about this in terms of community influence. According to one interviewee:

there was the absolute relief of meeting people who I identify with who just really struggle but have a go and make mistakes, shift around a bit and – to me it’s not always the most gratifying experience, and meeting those guys I find deeply motivating, and I just instantly feel a sense of community (Interviewee 22).

Other interviewees related similar feelings of motivation by individuals and in some instances gave concrete examples based on past experiences. For example, interviewees who strongly associated with Zoom sessions and a friend who practices said, “I like the guided better, cause I want to be, I want somebody to tell me” (Interviewee 08), and “someone giving me that appointment as such, that helps to motivate me to do it at that time” (Interviewee 06). Notably, the latter statements were somewhat ambiguous as to the extent of social identification, with some participants identifying as one of the broader mindfulness collective, without having a mindfulness group. Moreover, not all interviewees had stable motivation, reflecting the previous theme of fluidity of social identities. According to one interviewee, “there are times where I struggled more with my mental health when [that association] does feel stronger”, that is, “times when I’ve had more discussions with people […] or it’s kind of prompted me to read a bit more” (Interviewee 30). By contrast, when mental health was more stable, “I still read articles about that group, but I don’t feel a strong association with it, and I think that maybe as I’m talking about that, maybe that’s why I’m not always very proactive” (Interviewee 30).

#### Belief reinforcement.

While most interviewees reported positive beliefs about mindfulness, some of these reported experiences of belief reinforcement from other group members: “I suppose there is some kind of connection to have a discussion, learn more about it […] and find what else works for other people might work for me as well” (Interviewee 29). This interviewee was talking about conversations with a friend who also practiced, which was similar to another interviewee who explained, “when I have those discussions with [my friend] as well, it kind of resonates that yeah actually it’s a good thing for me to keep doing mindfulness” (Interviewee 6). This interviewee also shared similar identification with their mindfulness teacher. One interviewee even extended this identification to patients saying, “I always class myself as similar to one of the patients”, moreover, “that’s when I got hooked really and saw the results, yeah, so it was definitely, it was from seeing the results from other, the patients” (Interviewee 8). Speaking about the broader mental health group that also consisted of associations with mindfulness, finally:

I feel that it’s important for me to be part of that group and I like being part of that group because it encompasses a lot of things that interest me and it lets me know that other people experience similar things as well, have got similar experiences and are helped by similar things (Interviewee 30).

#### Emotional support.

A few interviewees found emotional support from mindfulness in-group members therapeutically important:

to talk to those people. You know, even if I said something and someone says, oh I completely agree with that, it’s then understanding, as well, okay I’m not the only one feeling like that or I’m not the only one going through the situation. […] It feels like you’ve got a bit of a safety in numbers, and you don’t feel completely out on a limb [slight laugh] (Interviewee 6).

Thus, focus resided with the conditions needed for engagement to thrive. At the same time, this interviewee acknowledged, “there’s times that you’re feeling like you need a bit of a boost actually sometimes going to someone who’s completely disjointed from your life normally can be quite beneficial”. One interviewee likened this boost/these conditions to sporting camaraderie:

a team like football or something, it’s like a shared goal at the end of it. You all experience the match a bit differently but actually at the end of it you all come together to go okay that was good, we enjoyed that, and we can reflect on it as a peer group (Interviewee 13).

Another interviewee discussed emotional support from their supervisor: “we do our own practice, yes, we will kind of share that with each other, and that becomes, so we identify that with each other and our own struggles with it” (Interviewee 31). Not everyone felt emotionally supported however:

I think if I was part of a group or I started I would feel like I was part of that, and then maybe would build relationships in that, but because my practice now is on my own when time permits I don’t feel like I connect (Interviewee 28).

### Dissonance about current levels of engagement

Some interviewees expressed discomfort at holding positive beliefs about mindfulness/members that did not match their own behaviours. Interviewee 8 who classed themself as similar to one of the patients said, “I’m a real advocate for [mindfulness] but I feel a complete fraud because I’m not using it as much as I feel I should. There’s a judgment [laughs]”. Interviewee 27 who stated that they have a lot of time for people who practice mindfulness reported, “It’s looking like I’m a disappointment to myself. That’s what that looks like. I know how beneficial [mindfulness] is to me, and I don’t do it”. Interviewee 28 on the one hand talked positively about the friend who taught mindfulness, saying, “when I’m with [that person] I think yeah I’d like to be a bit more like that”. On the other hand:

I know the benefits it would give me if I did it, so it’s so illogical that I’m like why can’t I now while I’m saying it, why can’t you fit it in, like literally why can’t I fit that in, but I just think I just don’t know how, I just don’t know how.

### Optimisation/ being aids doing

Around half of interviewees discussed ways in which mindfulness identities/ in-groups optimised work they do as part of NHS identities/ in-groups. Some interviewees chose to elaborate on benefits of normative behaviours associated with mindfulness identities, which was to some extent synonymous with being mindful, “like holding the space, holding difficult emotions without letting them overcome you” (Interviewee 18), whereas others gave accounts that were more explicitly group-related.

#### Practical uses with patients/ staff.

Some interviewees discussed practical benefits of being mindful at work, and these were split between patients and staff. When working with children as a speech and language therapist:

you have to be really focussed on their mouth shape, their resonance, lots of different aspects of their communication […] which is what mindfulness is, it’s almost like a hyper-focus on something to… this at the expense of other things, but I don’t mean that in a bad way, I mean it in a completely positive way […] where you, yeah. It’s almost like back to what I said at the beginning. You dull down the rest of the noise to allow you to, the main thing to shine bright and be as loud as possible (Interviewee 10).

When working as a nurse, mindfulness was useful, “to help you stay focussed in an assessment, whatever you’re doing” (Interviewee 1). Two physiotherapists chose to share skills with patients directly, for example, “advising my patients, I mean I don’t specifically suggest mindfulness to them, but it’s looking at different ways that they can help themselves” (Interviewee 06). Interviewees from psychological professions discussed potential benefits for staff, for example, “you can do these mindfulness practices within team meetings to help improve practice” (Interviewee 14). One doctor who felt that they had been put on a professional “pedestal” also wondered, “if actually if you were at a group with other people that it would help break down some of those barriers in the workplace. I think you would get more functional relationships out of it” (Interviewee 21).

#### Deeper appreciation of self/ practitioner.

Qualitatively different from the outwardly-focussed benefits elaborated in the subtheme above were the personal benefits of being mindful for oneself as an NHS worker. Several interviewees said that practicing mindfulness helped with self-appreciation and meeting personal needs:

Someone once said to me that the NHS will just take more and more and more [slight laugh], it’s like a needy child, the more you give the more it needs, and that’s really true. So you really have to be very boundaried, and mindfulness has really helped me to do that, to notice when I’m feeling exhausted and that I just have to stop (Interviewee 31).

Two participants spoke about mindfulness meeting the need to centre or ground, that is, “having that calm demeanour, centred approach and knowing that if something does trigger something you’ve got these methods and these techniques that you can use to bring yourself back down” (Interviewee 10). Likewise, “I think in terms of being like a reflective practitioner you need, it’s good to have that time to bring yourself back to yourself” (Interviewee 27). For some it was about developing resilience, not least to avoid “completely blowing up” (Interviewee 6).

### Aligning values and aspirations

About half of interviewees said that mindfulness and NHS identities/ in-groups aligned on values and aspirations. Half of these focussed on shared goals, and half expressed views about group compatibility that were contingent on prior culture and beliefs.

#### Shared goals and aspirations.

“You’re kind of working towards a common goal or a common aspiration,” explained Interviewee 12. Specifically, “you want to change lives and really improve the lives of people,” which aligned with “better[ing] ourselves” and the ability “to turn inwards and really be with ourselves and really tune in with ourselves”. The connotation here was of compassion for self and others. This interviewee concluded, “I guess they are both about change but that in mindfulness you are not striving for change, change will happen along the way”. Being and doing were thus seen to work synergistically, allowing one to be a problem solver, and do problem solving, both at the same time. Likewise, the remaining interviewees noted similarities between groups, such as the wish to understand, manage and show compassion for oneself and others. Seen from Interviewee 10’s perspective, for example, “it’s problem-solving. You’re trying to overcome a hurdle that either you’re aware of or you’re not aware of. So yeah, I think [those groups] are all interrelated, intertwined”.

#### Conditional on prior culture/ beliefs.

For some interviewees, mindfulness and NHS identities/ in-groups were deemed to be compatible but only when these were congruous with prior culture and beliefs:

[My service] was such a lovely place to work because I think probably a lot of people there did practice mindfulness and were very aware of it and were very aware of the importance of it and importance of allowing people to prioritise it as well. So yeah, I think they are very compatible (Interviewee 2).

This view was shared by Interviewee 8, who likewise believed in these groups’ compatibility while at the same time, “I think that people’s lack of understanding about mindfulness means it’s brought about that it conflicts, if that makes sense”. On a personal level, one interviewee felt drawn to mindfulness because of their therapy background, suggesting that mindfulness as an intervention was contingent on the beliefs one holds because of one’s profession (Interviewee 6). Conditionality was most obvious in the responses of three participants, one stating that mindfulness and NHS identities/ in-groups “can be compatible” (Interviewee 28), another stating they “should work together” (Interviewee 30), and least compellingly, “it’s not completely impossible to imagine” (Interviewee 21), when taking into account workplace and Western culture.

### Connection and disconnection

Mindfulness and NHS identities/ in-groups were perceived as complementary or conflicting depending on perceived connectivity between them for about half of interviewees. Again though, this was not necessarily a reflection of mindfulness practice levels. According to one interviewee, these identities were “really important and they all work with each other and rely on each other and, I think being a part of one identity makes the other ones better” (Interviewee 18). For another, “I think they’re connected, at times at odds but without one I can’t have the other” (Interviewee 13). Likewise, Interviewee 6 thought, “they all just complement each other really. It’s just that they’re everything that helps keeps your cup full”. However, other interviewees moved towards separation of these identities. Interviewee 12 said, “It’s a bit of a dividing line in terms of those two identities”, a view shared by Interviewee 30, who said, “being in the NHS is different to that part of me that is interested in mindfulness. […] I find that kind of merge, that pulling them together, quite difficult”. Interestingly, there was also an indication of movement over time:

my membership of the NHS tribe is not as absolute as it was because this other good thing happened. But in a way how funny that there was a price to pay in loss of membership of the NHS tribe (Interviewee 22).

### Culture clash

Broadly defined as the customs, beliefs and behaviours of a particular group, culture was seen to clash between mindfulness and NHS identities. “Just the culture of, and those pressures that come,” said Interviewee 31, later adding, “that’s the thing that needs to change in the NHS, that culture, that sense of really giving yourself that space”. Most interviewees, even those who discussed examples of synergy between identities/ in-groups, were confronted with service-level obstacles, primacy of work and/or unhelpful perceptions by others.

#### Service-level obstacles.

Service-level obstacles were wide-ranging. As argued by Interviewee 6, working on personal resilience is no salve for service-level problems with the NHS, “at the end of the day I’m being more resilient but the problem’s still there”, and “I’m still not achieving the service that I want to give to my patients”. Hence, mindfulness-group membership did not improve the service or professional self-efficacy. By contrast, Interviewee 8 thought there was a split between physical health and mental health sectors in how mindfulness is received, saying, “I think things like mindfulness […] and then the NHS groups that I’m in would fit together better. But there’s always, there’s a split”. Another interviewee flagged rote applications of mindfulness by NHS teams, that is, “mindfulness just being a task that you have to do at the beginning of a meeting, and it not being really embedded within the team” (Interviewee 14). Meanwhile, two participants identified service-level obstacles in good practice guidelines. Interviewee 24 thought that NICE guidelines overrepresented CBT, with one implication being that CBT training happens in work time but mindfulness training happens in one’s own time. Similarly, Interviewee 19 criticised lack of flexibility in mindfulness guidelines that mandate annual retreat attendance, again in one’s own time, since “part of that is my child/family commitments. Well I don’t have time to do the extra work but, it changes you know… because if you’re not meeting the guidelines, you’re not in the club”. This interviewee felt “removed” from some of the mindfulness community.

#### Primacy of work.

About half of interviewees thought their mindfulness identity should take a figurative back seat when navigating the NHS. “There’s definitely a time and a place for it,” argued Interviewee 1, emphasizing the need to focus on work when at work. This attitude was broadly shared by this contingent of interviewees, although reasons differed. First, there was a safety aspect, “you have to switch things off a little bit to get through something. So sometimes you’re just in a situation that feels quite dangerous” (Interviewee 18). There was also an ethical dimension when using mindfulness with service users, “I don’t have the right to impose that on service-users” (Interviewee 22), or by oneself when service users should take priority, “if I knew that it was my own meditation then I don’t know that I could do that with all good moral conscience” (Interviewee 31). This sentiment was echoed by Interviewee 29 who said, “I’ve got a service to provide […], and that’s my responsibility during my work time”. Hence, work identities were self-perceived as coming first, such that putting oneself first was even perceived negatively, “I’d feel a sense of, like I was taking the mick” (Interviewee 28).

#### Perception by others/ negatively distinctive.

Almost half of interviewees highlighted negative perceptions by others, with potential to see one’s own mindfulness identity as negatively distinctive. “Mindfulness, like I say, it’s incredibly non-judgemental,” postulated Interviewee 10, “where … in the NHS you do always get judged”. There was a perception of medical opposition to psychological interventions in one instance, “a lot of doctors, they’re kind of just a bit like…yeah I suppose dismissive of it, kind of patronising about it” (Interviewee 18). This view was supported by a doctor who conjectured on the place of mindfulness in GP practices, “people would perceive that as slightly odd I think, if you weren’t familiar with mindfulness. If you were, then the limitations of the small size of the business might be a problem” (Interviewee 21). However, negative perception was not reserved for medical professionals, as stated by a social worker, “I don’t see enough of that in the work that I do, those sort of people, the psychologists that I work with are very sort of risk focussed” (Interviewee 27). Another interviewee remarked, “there are groups out there, or individuals who think it’s a bit of old tosh” (Interviewee 30). Finally, the consequences of negative perceptions by others were not always explicit and could even erode motivation, for example:

if I was practicing [mindfulness] with people where it was forced upon them to practice and they didn’t really get the ethos or didn’t really want to be there, I think then that would be really disappointing because that might change the dynamics or my ability to be involved (Interviewee 28).

## Discussion

This thematic analysis sought to explore: 1) how NHS staff might socially identify with mindfulness groups; 2) how this might relate to psychological engagement; and 3) how cooccurring healthcare identities/in-groups might complement or clash. Corresponding themes were: **Richness** and **Fluidity** of social identities across multiple domains: Personal, Professional, Social contacts, Wider mindfulness, and Even wider wellbeing; **Positive boosts to engagement with mindfulness** from social identification, being Motivated by others, Belief reinforcement, and Emotional support, as well as **Dissonance about current levels of engagement**; and **Optimisation** from interaction of identities, such as Deeper appreciation of self/practitioner and Practical uses with patients/staff, coupled with **Aligning values and aspirations**, which included: Shared goals and aspirations, Conditional on prior culture/beliefs. There was a dual theme of **Connection and disconnection** on which perceptions of complementarity depended, but also **Culture clash**, comprised of Service-level obstacles, Primacy of work, and negatively distinctive Perception by others.

### Mindfulness and social identity

Several unexpected findings presented in **Richness of identities**. Rather than identifying with one’s mindfulness group or the broader mindfulness community, as might be expected, there was sometimes only a personal identification dimension, which included self-categorization of oneself as someone who practices mindfulness, as well as positivity and importance of this identity to one’s self concept, despite no explicit or conscious extension of this identification to other people. Contrary to the “positive emotional valuation of the relationship between self and ingroup” [34], this identification was nascent, presenting instead as the positive emotional valuation between self and mindfulness as an entity.

From a self-categorization perspective, we might say there was a salient lower-order category of mindfulness and “me”, and a non-salient higher-order category of mindfulness and “we”. For according to this theory [53], individuals can and do identify with both personal and social categories at varying levels of abstraction (rather than a simple binary, this is a hierarchy, with the lowest rung on the figurative ladder being “I”, ascending next to “we”, and next, higher still, to all human beings). These cognitive categorizations function dynamically in relation to changing contexts, with cognitive processes of self-stereotyping and depersonalisation also at play when re-defining the self and conforming to self-perceived norms. In the context of these interpersonal one-hour interviews between interviewee and interviewer, an individualistic rather than collectivistic identification with mindfulness sometimes prevailed.

Professional identification bridged this gap, such that interviewees identified with a professional entity that does include individuals, and the same went for Social contacts, and the Wider mindfulness community, even where individuals might be unknown. Perhaps most interesting was the bridge to others in the Even wider wellbeing community, for example identification with yoga groups, which included but were not limited to mindfulness. Not only does this finding support NICE guidelines [25], which specify that employers should improve wellbeing by providing access to mindfulness, yoga and meditation specifically, it underscores the quantitative evidence-base and provides a rich account of the meaning these practices can have for healthcare staff. Richness of identities also captured the tendency of interviewees to identify with multiple domains to varying degrees, for example when identifying personally and professionally. Multiple identification was a particularly interesting finding, since previous research looking outside of healthcare suggests that multiple identification predicts resilience [54], and wellbeing, but also that improvements in mental health can stem from increases in group compatibility [55]. Cruwys and colleagues refer to this phenomenon as “the psychological representation of one’s malleable social reality” (55, p.626), which in the present study saw mindfulness identities/ in-groups having multiple facets; this raises the question, might enhancement of complementary identity facets (within mindfulness, for example) lead to improvements in mental health and wellbeing as richness of identities increases, in keeping with malleable psychological representations? **Fluidity of social identities** also requires consideration in this regard, since some interviewees in the present study exhibited inconsistency in social identification with mindfulness identities/ in-groups. It is possible that less consistent/ most ambivalent responses had the least fixed/ most malleable identification, and therefore, that a contingent of healthcare staff could stand to benefit from strategies that help them to resolve complexities around how they feel about mindfulness, although this is speculation and would need to be explored in future research. Certainly, a contingent of interviewees did not extend identification beyond that of a personal relationship with mindfulness, and therefore, employers and trainers should acknowledge those participants for whom social identification played no explicit role. Catch-all initiatives that assume degrees of social identification in all staff are likely to meet with resistance. Indeed, several interviewees stressed the importance of not forcing mindfulness on staff for fear of putting them off, despite personally lauding the benefits of mindfulness practice. As found in the theme of **Culture clash** and specifically the subtheme of Negative perceptions, being in a group of people that are not fully engaged could just as easily put off participants who would otherwise be psychologically engaged.

### Social identification and psychological engagement with mindfulness

There were **Positive boosts to engagement** from social identification with mindfulness. Interviewees reported feeling Motivated by others, also reporting Belief reinforcement and Emotional support. These boosts mapped most closely onto Banerjee and colleagues’ [27] operational definition of engagement in regard to the first domain, 1) motivation to assign time, and fourth and fifth domains respectively, 4) believing in the potential benefits, and 5) being present to the therapeutic relationship. Remarkably, positive boosts to engagement from social identification were broadly similar to group effects found in prior studies that did not look specifically at social identification. In a meta-ethnography of studies by Malpass and colleagues [30], for example, motivation from the MBSR/MBCT group was helpful for patients’ continued practice when meeting challenges brought on by initial practice. Likewise, prior research emphasized the importance of “Belief in the Programme” [56] for participants engaging with MBCT; contrastingly, interviewees in the present study emphasized the importance of seeing results in others and the resonation of beliefs from discussion, i.e., that mindfulness helps, that mindfulness is a good thing. Numerous studies also cited emotional support, though often by separating this theme into its components, e.g., support from others, camaraderie, normalising, and the shared journey [57–60]. As highlighted by Banerjee and colleagues (28, p.1661), “The support of group and therapist has always emerged as a crucial theme in the experience of participating in MBIs”; hence rediscovery of this, and previous boosts, was not unexpected. What is unique in these instances is how social identification spoke to the mechanism of action for some aspects of psychological engagement in some participants (but not all). These distinctions bear potential implications for employers and trainers that may be seeking to optimise staff engagement with mindfulness.

**Dissonance about current levels of engagement** was also present. Consequently, it is important to stress that psychological engagement was not all positive. A wider point of this explorative study was manifest uncertainty around how much psychological engagement maps onto physical engagement, although views expressed in dissonance might help to explain why. In short, some interviewees expressed positive attitudes to mindfulness that were not always reflected in their practice, causing a minority of interviewees to feel negatively about disparity of beliefs and behaviours. Comparatively more interviewees saw links between social identification and psychological engagement even where they explicitly stated that physical engagement levels were currently low. Hence, voice was given to the (uneasy) state of flux that exists between psychological and physical engagement, and possibly, the sense of a tipping point in the change process cooccurring with positive views towards/ social identification with mindfulness. Such a finding is consistent with Prochaska and DiClemente’s [48] transtheoretical model of behaviour change, and more recent models of health behaviour, such as the Sussex Mindfulness Meditation Model (SuMMed) [61], which posits that establishing and maintaining a mindfulness practice is fraught with challenges characterised by stages that themselves represent a cognitive working through or threshold/tipping point to the next stage, for example, in the need for increasing self efficacy and outcome expectancies in order to move on. Certainly, it can be speculated that a loss of psychological engagement should lead to even less physical engagement with mindfulness practice. Conversely, more psychological engagement might dually serve to set the right conditions for physical engagement with mindfulness, which can result in action, inaction, and even negative self-schemas. At the same time, any optimisation sought from enhancing social identification could prove more complicated than a linear dose-response relationship where more social identification equals more engagement.

### Workplace identity and mindfulness identity

Cooccurring healthcare identities/ in-groups both complemented and clashed with mindfulness identities/ in-groups. Themes of **Optimisation**, **Aligning values and aspirations**, and **Culture clash** revolved around the dual theme of **Connection and disconnection**. Interestingly, this dual theme could be confused for a tautology, since for identities to complement they need to connect and vice versa for clashing. What was striking about this theme, however, and what lifted it above the level of description, was the degree of interrelation or compartmentalisation of identities rather than simply whether they worked well together or not. Given that multiple identification might predict resilience [54], and wellbeing, and that improvements in mental health might stem from increases in group compatibility [55], positive mental links or the absence of links between identities could mediate the success of any mindfulness-based initiatives that seek to enhance wellbeing in the workplace. Interestingly too, there was tentative support for the “loss of psychological “footing”” previously described by Haslam and colleagues [35] as participants adjust to new identities; one participant described how acquisition of the mindfulness identity led to distancing from the NHS identity, or gradual relegation from this in-group to the margins. However, more examples would be needed to confirm movement between identities over time.

It was expected that NHS and mindfulness identities might be defined by doing and being to varying degrees, and that NHS and mindfulness identities could work in synergy. Banerjee and colleagues (28, p.1658) found that participants that engaged with a mindfulness-based self-help intervention observed changes to their way of being, which in turn helped their engagement with the intervention, giving rise to the theme, “Becoming more mindful”. Although a similar theme was not produced in the present study of social identification, parallels were apparent in **Optimisation**, such that interviewees elaborated on perceived benefits of normative behaviours associated with mindfulness identities, i.e., Practical uses with patients/staff, as well as Deeper appreciation of self/practitioner. Analogous to the synergy of being mindful and doing mindfulness practice, there was synergy of being mindful and doing healthcare work. Unexpectedly, however, there was considerable polarity in attitudes towards compatibility, especially on the theme of **Culture clash**. Service-level obstacles, Primacy of work, and/or negative Perception by others all got in the way of what could otherwise be a synergistic relationship. Importantly, Tajfel and Turner [33] postulated that maintenance of identity depends on “favourable” comparisons but also distinctiveness from out-groups. Neither was evident here. Rather, there was evidence of unfavourable comparisons/negative distinctiveness for mindfulness identities within the NHS, though indeed, there was only minor evidence that mismatch led to disengagement from the unsatisfactory in-group or renewed efforts to increase its positive distinctiveness.

### Limitations

There are several limitations to the present study. First, findings are drawn from the broad body of data provided by interviewees who signed up to the study as part of a convenience sample. While this was a pragmatic necessity of the study owing to a relatively short timeframe, caution should be exercised when interpreting these findings as it is possible that participants self-selected on several attributes, such as interest in mindfulness, positivity towards mindfulness, or agreeableness to a transparent study of social identification. Second, interviewees’ retrospective reconstruction of experience in interviews could be unreliable, due to cognitive difficulties of memory recall. Third, based on the resulting number of interviewees per each profession/role that volunteered for this sub-study, considerably more interviewees would be needed to draw transferable conclusions on differences existing between professional groups, particularly seniority levels within these groups. Thematic saturation was the furthest from being reached for this element of the third research aim, or rather, this aspect of the research had the least information power. Findings from this study should be taken at face value – as interpreted meanings from the body of responses from a diverse sample of 20 healthcare staff. Only on this basis can we propose some degree of transferability of these findings to wider bodies such as the NHS and potentially other healthcare systems.

### Clinical implications and future research

Healthcare staff identified with mindfulness groups in diverse ways and to varying degrees, observing some positive boosts for some but not all aspects of psychological engagement. Yet, there was no explicit link between higher psychological engagement and physical engagement with mindfulness practice. Future research should consider experimental manipulation of identity facets for mindfulness (e.g., whether removing yoga from mindfulness decreases identification and psychological engagement), thus exploring possible effects on mental health and wellbeing, but also whether strength of social identification converts into improved physical engagement levels, in healthcare staff. In addition, experimental manipulations might be employed to partial out effects from any unmeasured variables that could also be accounting for psychological engagement, aside from social identification. Unmeasured variables might include self efficacy and outcome expectancies, as per the previously specified SuMMed model, or simply positivity towards mindfulness in the absence of social identification. Other factors could also be standing in the way of psychological engagement, including factors relating to work in the NHS, such as work pressures/ intensity, stress and burnout levels. For now, NHS employers and mindfulness trainers should consider deploying strategies that subtly enhance healthcare staff members’ sense of groupness or in-group, for example, when organising group training or granting access to interventions, perhaps via extended reflective discussions, buddying up of employees, or media platforms that encourage peer support. Such strategies might serve to capture that subgroup of participants for whom social identification is an important aspect of their psychological engagement. At the same time, caution should be exercised, especially with catch-all initiatives, to avoid alienating healthcare staff when seeking to optimise mindfulness in healthcare. Connection between mindfulness and NHS identities could be important for engagement with mindfulness in healthcare staff, but attitudes to connection may be polarised.

## Supporting information

S1 File Interview Schedule. (DOCX)
