# Supplementary material for: A qualitative exploration of NHS-staff social identification with mindfulness in-groups and engagement with mindful practices
Source: PLoS One. 2025 Oct 23;20(10):e0331196. doi: 10.1371/journal.pone.0331196 (PMC12548848; doi:10.1371/journal.pone.0331196)
Supplement: S1 File — (DOCX) [file pone.0331196.s001.docx]

**S1. Supporting information**

**Interview Schedule**

- Introduction/ welcome.
- *Reminder: Please be aware that your responses are being recorded to aid transcription. These recordings will be deleted on completion of the project.*
- *Reminder: Please do not mention yourself, any other person, or workplaces by name. This is to protect everyone’s anonymity.*

**Background/ NHS Groups:**

- Please can you tell me a little bit about your work background within the NHS?
  - Your job role or profession
  - Settings you’ve worked in
  - Teams you’ve worked in, if applicable
  - Your current level of expertise
- What inspired you to pursue the path you just described to me?
- You just mentioned several group memberships for yourself within the NHS (repeat back). Which of these group memberships do you view most positively and why?
  - Any others?
- Which of these group memberships is personally most important to you and why?
  - Any others?

**Mindfulness and Psychological Engagement (Cycle of Change):**

- How did you first hear about mindfulness? (i.e., what it is, what it’s good for, etc..) (*pre-contemplation*)
- What first inspired you to give mindfulness a go? (*contemplation*)
  - Were you trying to change something?
  - Did someone else recommend it?
  - Was there more than one of you having a go?
- How did you prepare to start practicing? (*preparation*)
  - Did you join a group, download an app, talk to friends? etc.
- How would you describe your experience of mindfulness so far? (*action*)
  - In terms of motivation to assign time at work or home?
  - How about your intention to practice at work or home?
  - Or your commitment to being more mindful in daily life?
  - Your belief in the potential benefits of mindfulness?
  - Do you see any kind of therapeutic relationship there?
- How would you feel about being part of a mindfulness group going forward? (*maintenance*)
- Why stop? (*termination*)
  - Why did you/why might you?

**Mindfulness, Social Comparisons and Social Identification**

- What is your impression of other people who practice mindfulness?
  - Alternatively, what kind of individuals do you think practice mindfulness?
  - Where do you see them in society?
- What is your impression of mindfulness teachers? (e.g., In-class, teacher who wrote the book, or the voice on the audio guide etc.)
  - Alternatively, what kind of individuals do you think teach mindfulness?
  - Where do you see them in society?
- What would you say about mindfulness to someone who was considering it?
- Do you personally identify with mindfulness groups, movements or group members in any way? (e.g., the group you practice with, Headspace members, friends who practice mindfulness or the larger mindfulness community)
  - How does this association make you feel?
  - How important is being part of this mindfulness group or the wider mindfulness community for you?
  - Has this changed since switching from in-person to virtual groups? (if relevant)
- How does membership of a mindfulness group or community compare with those memberships you described for the NHS?
  - In your case, you previously said… (repeat back)
  - Do you think they are compatible?
  - Tell me what ways you think they work well together
  - Tell me what ways they do not work so well together
- What other group memberships outside of work or mindfulness are positive and/or important to you?
  - If so, do these fit well with your NHS and mindfulness group memberships?

**Thank you!**
